# Association between plasma fluorescent oxidation products and erectile dysfunction: A prospective study

**DOI:** 10.1186/s12894-015-0083-9

**Published:** 2015-08-14

**Authors:** Shuman Yang, Edward Giovannucci, Bruce Bracken, Shuk-Mei Ho, Tianying Wu

**Affiliations:** Division of Epidemiology and Biostatistics, Department of Environmental Health, University of Cincinnati Medical Center, Kettering Complex, 3223 Eden Ave, Cincinnati, OH USA 45267-0056; Department of Surgery, University of Cincinnati Medical Center, Cincinnati, OH USA; Division of Environmental Genetics and Molecular Toxicology, Cincinnati, OH USA; Center for Environmental Genetics, University of Cincinnati Medical Center, Cincinnati, OH USA; Departments of Nutrition and Epidemiology, Harvard School of Public Health, Boston, MA USA; The Channing Division of Network Medicine, Department of Medicine, Brigham and Women’s Hospital, Harvard Medical School, Boston, MA USA

## Abstract

**Background:**

Existing epidemiological studies of the association between oxidative stress and erectile dysfunction (ED) are sparse and inconclusive, which is likely due to cross-sectional design and small sample size. Therefore, we investigated the association between biomarkers of oxidative stress and ED in prospective setting among a relatively large sample size of men.

**Methods:**

We conducted the prospective study among 917 men ages between 47 and 80 years at the time of blood draw, which is a part of nested prospective case–control study of prostate cancer in the Health Professionals Follow-up Study. Plasma fluorescent oxidation products (FlOPs), a global biomarker for oxidative stress, were measured at three excitation/emission wavelengths (360/420 nm named as FlOP_360; 320/420 nm named as FlOP_320 and 400/475 nm named as FlOP_400).

**Results:**

Approximately 35 % of men developed ED during follow-up. We did not find an independent association between FlOP_360, FlOP_320, FlOP_400 and risk of ED in the multivariable adjusted model (Tertile 3 vs. tertile 1: odds ratio [OR] = 0.90, 95 % confidence interval [CI] = 0.61-1.34, *P*_trend_ = 0.54 for FlOP_360; OR = 0.73, 95 % CI = 0.49-1.07, *P*_trend_ = 0.27 for FlOP_320; and OR = 0.98, 95 % CI = 0.66-1.45, *P*_trend_ = 0.72 for FlOP_400). Further analysis of the association between FlOPs and ED in the fasting samples or controls only (free of prostate cancer incidence) did not change the results appreciably.

**Conclusions:**

Plasma FlOPs were not associated with the risk of ED, suggesting oxidative stress may not be an independent risk factor for ED.

**Electronic supplementary material:**

The online version of this article (doi:10.1186/s12894-015-0083-9) contains supplementary material, which is available to authorized users.

## Background

Oxidative stress reflects an imbalance between systemic levels of reactive oxygen species (ROSs) and host antioxidant defense systems that are able to counteract (detoxify) these ROSs. Insufficient antioxidant defense systems against ROSs can result in damage to proteins, lipids and DNA in cells and organs in humans. High level of oxidative stress is an important risk factor for many prevalent diseases including cardiovascular disease, breast cancer and reduced renal function [1–3].

The level of plasma fluorescent oxidation products (FlOPs) is a reliable and convenient approach to assess circulating oxidative stress in large epidemiological studies. One advantage of this marker as compared to other traditional specific oxidation markers (i.e., F2-isoprostanes and malondialdehyde) is that FLOP assay reflects oxidation pathways from multiple sources including lipid, protein and DNA [4, 5], whereas traditional specific oxidation markers reflect only a portion of oxidative stress. In large observational studies, we found that the level of plasma FlOPs is increased with hypertension, smoking and reduced renal function as defined by reduced levels of glomerular filtration rate [1, 3, 5, 6]. Furthermore, we have documented that the FlOP assay is robust in epidemiologic and clinical setting in which the collection and processing of blood samples cannot be well-controlled. We have found that FlOPs are stable in the blood samples with delayed processing up to 48 h at 4 °C, stable for more than 10 years in plasma samples in liquid-nitrogen freezers, and highly reproducible over 1–2 year among the same individuals [5, 7].

The association between oxidative stress and erectile dysfunction (ED) is sparse and inconclusive, which is likely due to cross-sectional design and small sample size. It is well known that oxidative stress plays an important role in the development of atherosclerotic diseases [8, 9]. Atherosclerosis reduces cavernosal blood flow, leading to vasculogenic ED [10, 11]. However, existing epidemiological studies of the association between oxidative stress and ED either had small sample size (N ≤ 60) or were cross-sectional [12–14]. Further, none of above studies adjusted for important potential confounders such as diabetes, hypertension and cigarette smoking which are the risk factors for ED and are important determinants of oxidative stress [5, 15, 16]. Large and prospective studies are warranted to examine the independent relationship between oxidative stress and ED. Therefore, we investigated the association between plasma FlOPs and ED in prospective settings among a relatively large sample size of men.

## Methods

### Study participants and blood collection

**Fig. 1:**
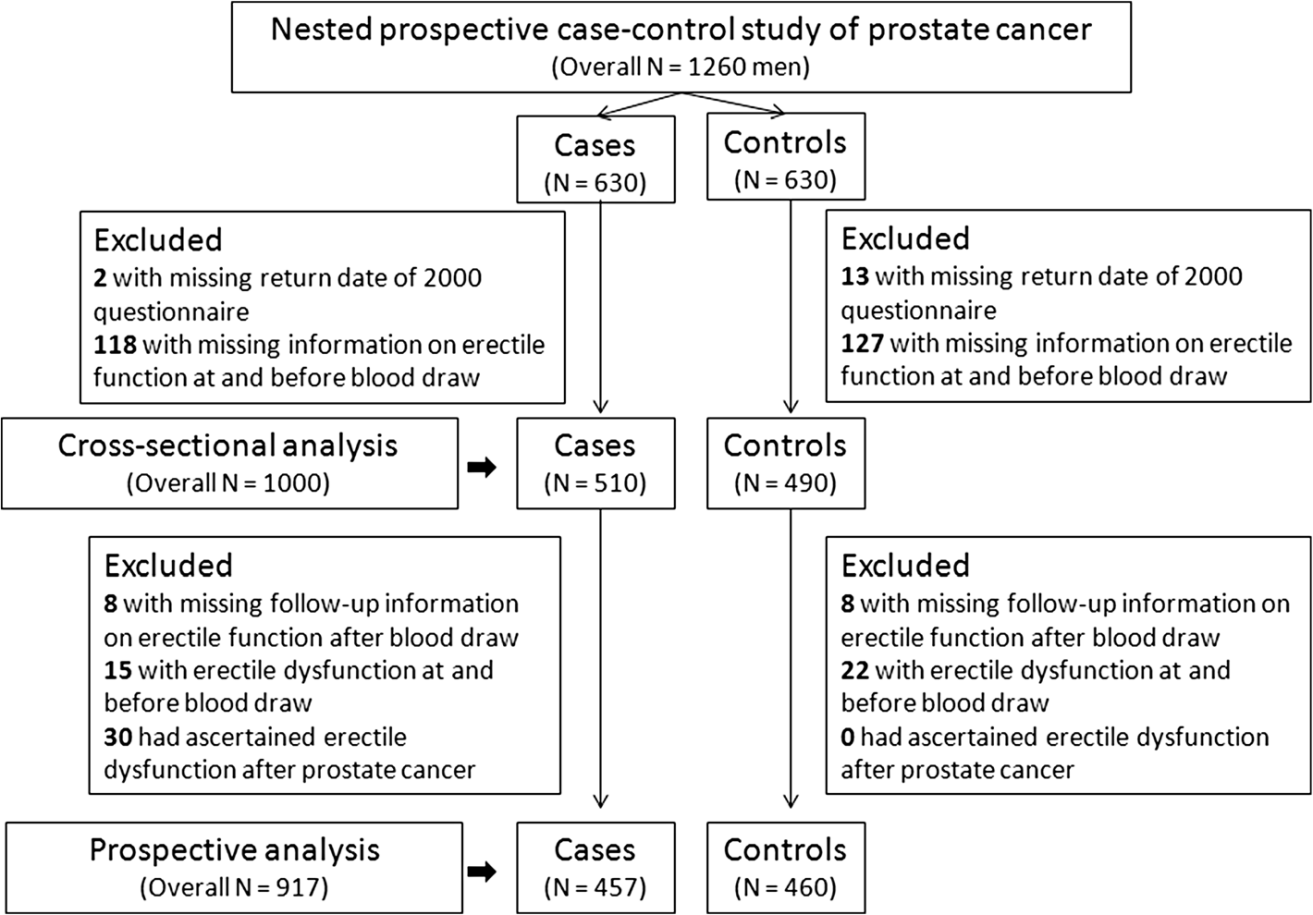
Flowchart of the participants excluded from the nested prospective case–control study of prostate cancer in the Health Professional Follow-up Study 1993–2001

The Health Professionals Follow-up Study (HPFS) initiated in 1986 is an ongoing prospective study of 51,529 men. Between 1993 and 1995, blood collection kits were sent to participants and 18,140 men returned specimens on ice by using an overnight courier. All returned blood samples were processed within 36 h after blood draw and stored in liquid nitrogen freezers. Based on the participants who donated blood samples, a 1:1 matched nested prospective case–control study of prostate cancer was performed from the time of blood draw [17]. All participants were free of diagnosed cardiovascular diseases and cancers at the time of blood draw. After excluding the ineligible participants (Fig. 1), we finally included 917 men ages between 47 and 80 years (median = 62 years) at the time of blood draw in the prospective study. Among 917 men, 457 and 460 men were subsequent incident prostate cancer cases and controls, respectively. Written informed consent was obtained from all participants. This investigation was approved by Institutional Review Board of the Brigham and Women’s Hospital, the Harvard School of Public Health and the University of Cincinnati.

### Measurement of FlOPs

#### Assay procedure

We measured plasma FlOPs using previously described procedures [5]. In brief, plasma was extracted with ethanol/ether (3:1, v/v) and centrifuged to obtain supernatant. We measured fluorescence of the supernatant at three wavelengths (360/420 nm [excitation/emission] named as FlOP_360, 320/420 nm named as FlOP_320 and 400/475 nm named as FlOP_400). FlOP_360 represents the interaction between lipid oxidation products and proteins, DNA and carbohydrates. FlOP_320 can be produced when oxidation products such as lipid hydroperoxides, aldehydes, and ketones react with DNA in the presence of metals, and FlOP_360 reflects the interaction between malondialdehyde, proteins and phospholipids [18]. The within-run average coefficient of variations for FlOP measurements were < 13 %.

#### Assay stability in blood samples with delayed processing

All blood samples were processed within 36 h after receiving the samples. The delay in processing blood samples up to 36 h appeared to have minimal influence on the measurement of FlOPs. The overall intraclass correlation coefficients (ICCs) of FlOPs were all greater than 0.95 in the shorter- (0 to 24 h) and longer-delayed processing (0 to 36 h) [7].

#### Assay between- and within- person reproducibility

We conducted a pilot study in 40 participants who donated two blood samples from the Nurse Health Study. After adjusting for fasting status, the ICC of the between- and within- person variations of the FlOPs for repeated measurements over 1.4 year apart (range: 0.8-2.2 years) was 0.44 for FlOP_360, 0.55 for FlOP_320, and 0.70 for FlOP_400 [3].

### ED Ascertainment

Between 2000 and 2001, a recall questionnaire in HPFS participants was initiated to rate their ability to have and maintain an erection sufficient for sexual intercourse. The question was “Please rate your ability (without treatment) to have and maintain an erection good enough for intercourse for the following time periods:”. Time periods included “before 1986”, “1986-1989”, “1990-1994”, “1995 or later” and “in the last 3 months”. Responses of the questionnaire included “very poor”, “poor”, “fair”, “good” and “very good”. ED was defined if participants answered “very poor” or “poor”. Any ED cases ascertained clearly after blood draw (1993–1995) were considered incident ED. If year of blood draw and year of ED ascertainment may be overlapping (i.e., year of blood draw was 1995 and period of ED ascertainment was ‘1995 or later’), these ED cases were not defined as incident ED. If the ED cases were not defined as incidence above, these ED cases were used in the cross-sectional analysis only (Fig. 1). Regardless ED cases recovered at a later time or not, they were defined as ED cases in the current study. Whether these cases were included in the cross-sectional or prospective analysis was dependent on the time period of ED ascertainment.

### Statistical analyses

To make a comprehensive understanding on relationship between plasma FlOPs and ED, we performed both prospective and cross-sectional analysis (Fig. 1). In the prospective analysis, we analyzed the association between plasma FlOPs and incidence of ED with logistic regression model, but not with Cox proportional hazard model as we were not exactly sure the year of incident EDThe risk of incident ED in lowest tertile of FlOPs was compared with that in the second and third tertile of FlOPs. The covariates included in the prospective analysis were age (continuous), body mass index (BMI, continuous), alcohol intake (in quartiles: < 0.9, ≥ 0.9 and < 6.6, ≥ 6.6 and < 17.2, ≥ 17.2 g/day), physical activity (in quartiles: < 13.85, ≥ 13.85 and < 28.9, ≥ 28.9 and < 51.95, ≥ 51.95 MET-hours/week), Caucasian (yes/no), fasting hours (continuous), benign prostatic hyperplasia with surgery (yes/no), history of hypertension (yes/no), history of diabetes (yes/no), smoking status (current smokers, past smokers and non-smokers), month of blood draw (in seasons: Spring [March, April and May], Summer [June, July and August], Fall [September, October and November], Winter [December, January and February]) and year of blood draw (1993, 1994 and 1995). To further rule out the potential confounding by fasting status and subclinical factors of prostate cancer incidence, we also examined the association between FlOPs and ED in fasting samples and controls only (free of prostate cancer incidence), respectively. In the cross-sectional analysis, we examined the association between plasma FlOPs and ED at baseline with logistic regression model. The covariates included in the cross-sectional analysis were same as the prospective analysis. All analyses were performed with Statistical Analysis System (Version 9, SAS Institute Inc., Cary, NC).

## Results

### Baseline characteristics according to plasma FlOP levels

In the prospective analysis, higher levels of plasma FlOP_360, FlOP_320 and FlOP_400 were associated with older age, greater alcohol intake, lower proportion of men who had fasted ≥ 8 h before blood draw and great proportion of current and past smokers (Table 1). Higher levels of FlOP_360 were correlated with greater proportion of history of hypertension. Higher levels of FlOP_400 were correlated with greater BMI. When similar analysis was performed in prostate cancer cases and controls separately, the relationship between baseline characteristics and FlOPs in either group was similar to that in the overall samples (Additional file 1: Table S1 and Additional file 2: Table S2). Moreover, the relationship between baseline characteristics in propective analysis (Table 1) was comparable to that in cross-sectional analysis (Additional file 3: Table S3).

**Table 1:** Baseline characteristics according to tertiles of plasma fluorescent oxidation products (FlOPs) (*N* = 917): prospective analysis in the Health Professional Follow-up Study, 1993-1995

Variables	FlOP_360			FlOP_320			FlOP_400		
Tertile	1	2	3	1	2	3	1	2	3
Range (FI/ml)	< 184	≥ 184; < 233	≥ 233	< 356	≥ 356; < 524	≥ 524	< 49.1	≥ 49.1; < 62.6	≥ 62.6
N	305	306	306	305	306	306	305	306	306
Age (years)	**61.1**	**61.9**	**62.3**	**60.4**	**62.2**	**62.6**	**60.7**	**62.3**	**62.2**
Body mass index (kg/m^2^)	25.8	25.8	25.7	25.5	25.9	25.9	**25.4**	**25.9**	**26.0**
Alcohol intake (g/day)^a^	**2.9**	**8.2**	**10.0**	**3.5**	**8.8**	**8.6**	**2.4**	**8.2**	**10.4**
Physical activity (MET-hours/week)^a^	26.0	33.6	29.1	27.9	34.2	26.5	28.3	29.8	28.1
Caucasians (%)	92	92	95	91	95	93	93	91	95
Fasting status (≥ 8 h; %)	**76.4**	**56.2**	**50.3**	**75.7**	**56.5**	**50.7**	**71.2**	**59.5**	**52.3**
History of BPH with surgery (%)	1.6	3.6	3.3	1.3	3.3	3.9	2.0	3.6	2.9
History of hypertension (%)	**23.3**	**23.9**	**31.4**	20.3	30.4	27.8	21.3	31.1	26.1
History of diabetes (%)	3.6	2.6	2.9	2.6	3.3	3.3	2.3	3.9	2.9
Current smokers (%)	**1.1**	**6.7**	**13.0**	**1.1**	**9.6**	**9.4**	**1.1**	**4.2**	**16.5**
Past smokers (%)	**37.8**	**49.8**	**60.8**	**38.6**	**55.4**	**54.4**	**35.1**	**50.9**	**62.7**

Variables with normal distribution are shown in mean, unless otherwise specified

^a^Variables with skew distribution are shown in median

Abbreviations: FlOP = Fluorescent oxidation products, FI = Fluorescent intensity unites, MET = Metabolic equivalent, BPH = Benign prostatic hyperplasia

Bold-faced values indicate statistically significance at *P* < 0.05 across tertiles of FlOPs

#### Association between plasma FlOPs and ED

In the prospective analysis, 35 % (*N* = 323) of men were identified having incident ED after blood draw. Although the proportion of incident ED appeared to be higher among men with higher levels of FlOPs (Table 2), we did not find an independent association between FlOP_360, FlOP_320, FlOP_400 and risk of ED in the multivariable adjusted model (Tertile 3 vs. tertile 1: OR = 0.90, 95 % CI = 0.61-1.34, *P*_trend_ = 0.54 for FlOP_360; OR = 0.73, 95 % CI = 0.49-1.07, *P*_trend_ = 0.27 for FlOP_320; and OR = 0.98, 95 % CI = 0.66-1.45, *P*_trend_ = 0.72 for FlOP_400).

**Table 2:** Association between plasma fluorescent oxidation products (FlOPs) and erectile dysfunction in prostate cancer cases (*N* =457) and controls (*N* = 460): prospective analysis in the Health Professional Follow-up Study, 1993-2001

Tertile	1	2	3	*P* for trend
Variables	FlOP_360			
Range (FI/ml)	< 184	≥ 184; < 233	≥ 233	---
N	305	306	306	---
Median (FI/ml)	160	207	280	---
Erectile dysfunction incidence (n, %)	100 (33 %)	112 (37 %)	111 (36 %)	---
Age adjusted	1 (ref)	1.13 (0.79, 1.61)	1.07 (0.75, 1.52)	0.84
Multivariable adjusted^a^	1 (ref)	1.03 (0.71, 1.51)	0.90 (0.61, 1.34)	0.54
	FlOP_320			
Range (FI/ml)	< 356	≥ 356; < 524	≥ 524	---
N	305	306	306	---
Median (FI/ml)	302	410	1837	---
Erectile dysfunction incidence (n, %)	102 (33 %)	114 (37 %)	107 (35 %)	---
Age adjusted	1 (ref)	1.00 (0.70, 1.42)	0.86 (0.60, 1.23)	0.30
Multivariable adjusted^a^	1 (ref)	0.75 (0.51, 1.11)	0.73 (0.49, 1.07)	0.27
	FlOP_400			
Range (FI/ml)	< 49.1	≥ 49.1; < 62.6	≥ 62.6	---
N	305	306	306	---
Median (FI/ml)	44.0	55.1	72.6	---
Erectile dysfunction incidence (n, %)	100 (33 %)	99 (32 %)	124 (41 %)	---
Age adjusted	1 (ref)	0.82 (0.57, 1.17)	1.24 (0.87, 1.77)	0.14
Multivariable adjusted^a^	1 (ref)	0.67 (0.45, 0.99)	0.98 (0.66, 1.45)	0.72

Values are odds ratio (95 % confidence interval), unless otherwise specified. FI = Fluorescent intensity units

^a^Risk factors include age (continuous), body mass index (continuous), alcohol intake (in quartiles: < 0.88, ≥ 0.88 and < 5.58, ≥ 5.58 and < 16.38, ≥ 16.38 g/day), physical activity (in quartiles: < 15.6, ≥ 15.6and < 30.6, ≥ 30.6 and < 56.9, ≥ 56.9 MET-hours/week), Caucasian (yes/no), fasting hours (continuous), benign prostatic hyperplasia with surgery (yes/no), history of hypertension (yes/no), history of diabetes (yes/no), smoking status (current smokers, past smokers and non-smokers), month of blood draw (in seasons: Spring [March, April and May], Summer [June, July and August], Fall [September, October and November], Winter [December, January and February]) and year of blood draw (1993, 1994 and 1995)

Further analysis of the association between FlOPs and ED in the fasting samples (≥ 8 h) did not change the results appreciably (Tertile 3 vs. tertile 1: OR = 0.91, 95 % CI = 0.56-1.47, *P*_trend_ = 0.69 for FlOP_360; OR = 0.75, 95 % CI = 0.46-1.21, *P*_trend_ = 0.52 for FlOP_320; and OR = 0.83, 95 % CI = 0.51-1.35, *P*_trend_ = 0.53 for FlOP_400).

When we analyzed the relationship between plasma FlOPs and risk of ED in controls only (free of prostate cancer incidence), levels of FlOP_360, FlOP_320 and FlOP_400 were again not associated with increased risk of ED (Table 3).

**Table 3:** Association between plasma fluorescent oxidation products (FlOPs) and erectile dysfunction in controls only (*N* = 460): prospective analysis in the Health Professional Follow-up Study, 1993-2001

Tertile	1	2	3	*P* for trend
Variables	FlOP_360			
Range (FI/ml)	< 184	≥ 184; < 233	≥ 233	---
N	164	144	152	---
Median (FI/ml)	159	206	279	---
Erectile dysfunction incidence (n, %)	55 (34 %)	51 (35 %)	51 (32 %)	---
Age adjusted	1 (ref)	1.06 (0.64, 1.75)	0.90 (0.54, 1.48)	0.59
Multivariable adjusted^a^	1 (ref)	1.02 (0.58, 1.79)	0.83 (0.47, 1.47)	0.46
	FlOP_320			
Range (FI/ml)	< 356	≥ 356; < 524	≥ 524	---
N	160	142	158	---
Median (FI/ml)	297	411	1858	---
Erectile dysfunction incidence (n, %)	52 (33 %)	53 (37 %)	52 (33 %)	---
Age adjusted	1 (ref)	0.96 (0.57, 1.60)	0.73 (0.44, 1.22)	0.17
Multivariable adjusted^a^	1 (ref)	0.75 (0.42, 1.35)	0.55 (0.31, 0.99)	0.10
	FlOP_400			
Range (FI/ml)	< 49.1	≥ 49.1; < 62.6	≥ 62.6	---
N	150	160	150	---
Median (FI/ml)	43.4	54.9	73.3	---
Erectile dysfunction incidence (n, %)	50 (33 %)	48 (31 %)	59 (39 %)	---
Age adjusted	1 (ref)	0.77 (0.46, 1.28)	1.23 (0.74, 2.03)	0.29
Multivariable adjusted^a^	1 (ref)	0.72 (0.41, 1.28)	1.07 (0.60, 1.88)	0.68

Values are odds ratio (95 % confidence interval), unless otherwise specified. FI = Fluorescent intensity units

^a^Risk factors include age (continuous), body mass index (continuous), alcohol intake (in quartiles: < 0.88, ≥ 0.88 and < 5.58, ≥ 5.58 and < 16.38, ≥ 16.38 g/day), physical activity (in quartiles: < 15.6, ≥ 15.6and < 30.6, ≥ 30.6 and < 56.9, ≥ 56.9 MET-hours/week), Caucasian (yes/no), fasting hours (continuous), benign prostatic hyperplasia with surgery (yes/no), history of hypertension (yes/no), history of diabetes (yes/no), smoking status (current smokers, past smokers and non-smokers), month of blood draw (in seasons: Spring [March, April and May], Summer [June, July and August], Fall [September, October and November], Winter [December, January and February]) and year of blood draw (1993, 1994 and 1995)

In the cross-sectional analysis, we found 3.7 % (*N* = 37) of men with ED at the time of blood draw. Higher levels of FlOP_360 were associated with increased risk of baseline ED (Tertile 3 vs. tertile 1: odds ratio [OR] = 2.68, 95 % confidence interval [CI] = 1.01-7.12), and the relationship had a significant trend (*P*_trend_ = 0.03). However, higher levels of FlOP_320 and FlOP_400 were not associated with baseline ED (Additional file 4: Table S4).

### Discussion

To our knowledge, this is the first study that comprehensively assessed the association between oxidative stress and ED in both prospective and cross-sectional designs. Although higher levels of FlOP_360 were associated with increased risk of ED in the cross-sectional design, none of FlOP_360, FlOP_320 or FlOP_400 was associated with incidence of ED in the prospective design. Since the relationship between biomarkers of oxidative stress and ED is largely derived from animal models [10], the results of our study have challenged the traditional understandings on the independent detrimental effects of oxidative stress on ED in human.

We only found FlOP_360, but not FlOP_320 or FlOP_400, was positively associated with ED in cross-sectional design. Several reasons may be responsible: First, we cannot fully exclude a possibility of false-positive findings due to the fact that cross-sectional analysis had a small number of ED cases (N = 37) and only one type of FlOPs (FlOP_360 only) was associated with ED. Second, because of the cross-sectional design, the positive relationship between FlOP_360 and ED in cross-sectional design may be due to ED-related diseases that are correlated with oxidative stress. Certainly, further studies are warranted to confirm this.

In contrast to the positive association between FlOP_360 and ED in the cross-sectional study, we did not show consistent evidence of a positive association between oxidative stress and risk of ED in the prospective design, which had approximately 9 times as many cases as cross-sectional analysis. Besides the much larger number of cases, the prospective design limited reverse causation. However, it is possible that the underlying mechanism of oxidative stress on ED was not due to a global oxidative burden but a specific type of ROSs. There is animal and tissue evidence suggesting that superoxide with nitric oxide can result in acute impairment of cavernosal relaxation but also long-term penile vasculopathy [19–21].

The study has several limitations. First, a single measurement of FlOPs may not accurately reflect the average levels of the biomarker over a prolonged period of time. However, we have assessed their reproducibility over approximately a one-year period, and high ICCs suggest that this marker can be used as a marker for chronic exposure. Second, since we only ascertained ED via a single self-reported questionnaire on ED onset during approximately 7 years after blood draw, we cannot exclude the recall bias as a possible explanation of our results. Furthermore, our assessment of ED has not been validated; however, we note that a prior report using these data [22] were consistent with what has been found in other studies [15, 23]. Nonetheless, these two methodological weaknesses regarding the ascertainment of ED may lead to misclassification between incident ED and healthy controls. Third, our study is limited because the specific oxidative stress level at penile site was not available. Fourth, as our study only included history of BPH with surgery, some BPH cases with less severe clinical conditions might not be included. Fifth, the bias due to other known (i.e., duration of hypertension and smoking) or unknown residual confounding factors that are related to plasma FlOPs and ED is likely present. Sixth, our study was derived from another study designed to study prostate cancer but not ED. The sampling of that nested case–control study may introduce biases that affected our study and may limit the generation of our results. All the above limitations may cause the null association between plasma FlOPs and incident ED.

The strength of the study is that it contained relatively large sample size of men and incident EDs. In addition, as mentioned above, the prospective study design is a better design than cross-sectional design to reduce the possibility of the reverse causation between oxidative stress and ED, although an effect of subclinical disease on biomarkers cannot be excluded.

## Conclusions

In conclusion, we found no overall association between plasma FlOPs and risk of ED. This has raised an important message that systemic oxidative stress markers overall may not be a relevant measure for assessing the risk of incident ED. Therefore, the necessity of oxidative stress measurement in the risk of ED assessment is questionable. Since this is the first prospective study on biomarkers of global oxidation only, further investigation of other biomarkers of oxidative stress in relation to ED is warranted.

## Additional files

Additional file 1: Table S1. Baseline characteristics according to tertiles of plasma fluorescent oxidation products in the Health Professional Follow-up Study in prostate cancer cases (*N* = 457), 1993–1995. (DOCX 14 kb) Additional file 2: Table S2. Baseline characteristics according to tertiles of plasma fluorescent oxidation products in the Health Professional Follow-up Study in controls (*N* = 460), 1993–1995. (DOCX 15 kb) Additional file 3: Table S3. Baseline characteristics according to tertiles of plasma fluorescent oxidation products (FlOPs) (*N* = 1,000): cross-sectional analysis in the Health Professional Follow-up Study, 1993–1995. (DOCX 13 kb) Additional file 4: Table S4. Association between plasma fluorescent oxidation products (FlOPs) and erectile dysfunction (*N* = 1,000): cross-sectional analysis in the Health Professional Follow-up Study, 1993–1995. (DOCX 15 kb)
