# Supplementary material for: Association between plasma fluorescent oxidation products and erectile dysfunction: A prospective study
Source: BMC Urol. 2015 Aug 14;15:85. doi: 10.1186/s12894-015-0083-9 (PMC4536733; doi:10.1186/s12894-015-0083-9)
Supplement: Additional file 1: Table S1. — Baseline characteristics according to tertiles of plasma fluorescent oxidation products in the Health Professional Follow-up Study in prostate cancer cases (N = 457), 1993–1995. (DOCX 14 kb) [file 12894_2015_83_MOESM1_ESM.docx]

**Additional file 1: Table S1. Baseline characteristics according to tertiles of plasma fluorescent oxidation products in the Health Professional Follow-up Study in prostate cancer cases (N = 457), 1993-1995**

| **Variables** | **FlOP_360** | | | **FlOP_320** | | | **FlOP_400** | | |
| --- | --- | --- | --- | --- | --- | --- | --- | --- | --- |
| Tertile | 1 | 2 | 3 | 1 | 2 | 3 | 1 | 2 | 3 |
| Range (FI/ml) | < 184 | ≥ 184;  < 233 | ≥ 233 | < 356 | ≥ 356;  < 524 | ≥ 524 | < 49.1 | ≥ 49.1;  < 62.6 | ≥ 62.6 |
| N | 141 | 162 | 154 | 145 | 164 | 148 | 155 | 146 | 156 |
| Age (years) | **60.8** | **61.9** | **62.0** | 60.7 | 61.9 | 62.1 | **60.0** | **62.6** | **62.2** |
| Body mass index (kg/m^2^) | 25.8 | 25.7 | 25.4 | 25.5 | 25.9 | 25.4 | 25.2 | 25.9 | 25.8 |
| Alcohol intake (g/day)* | **3.9** | **7.3** | **10.3** | 4.6 | 7.8 | 8.8 | **2.5** | **9.0** | **10.6** |
| Physical activity (MET-hours/week)* | 21.4 | 30.5 | 28.2 | 25.6 | 31.1 | 24.5 | 28.3 | 26.1 | 28.7 |
| Caucasians (%) | 96 | 94 | 95 | **96** | **97** | **92** | 94 | 96 | 95 |
| Fasting status (≥ 8 hours; %) | **74.5** | **53.1** | **48.7** | **73.1** | **55.5** | **46.6** | **67.7** | **58.9** | **48.1** |
| History of BPH with surgery (%) | 0.7 | 3.1 | 3.3 | 0.7 | 3.7 | 2.7 | 1.3 | 3.4 | 2.6 |
| History of hypertension (%) | 20.6 | 22.2 | 27.3 | 17.9 | 27.4 | 24.3 | 19.4 | 28.1 | 23.1 |
| History of diabetes (%) | 3.6 | 2.5 | 1.3 | 3.5 | 2.4 | 1.4 | 2.6 | 4.1 | 0.6 |
| Current smokers (%) | **0** | **7.1** | **13.2** | **0** | **9.2** | **10.7** | **0.9** | **1.5** | **19.4** |
| Past smokers (%) | **33.1** | **47.7** | **57.6** | 33.8 | 54.9 | 49.2 | **25.5** | **52.9** | **61.2** |

Variables with normal distribution are shown in mean, unless otherwise specified. *Variables with skew distribution are shown in median. Abbreviations: FlOP = Fluorescent oxidation products, FI = Fluorescent intensity units, MET = Metabolic equivalent, BPH = Benign prostatic hyperplasia.

Bold-faced values indicate statistically significance at *P* < 0.05 across tertiles of FlOPs.
