# Supplementary material for: Association between plasma fluorescent oxidation products and erectile dysfunction: A prospective study
Source: BMC Urol. 2015 Aug 14;15:85. doi: 10.1186/s12894-015-0083-9 (PMC4536733; doi:10.1186/s12894-015-0083-9)
Supplement: Additional file 2: Table S2. — Baseline characteristics according to tertiles of plasma fluorescent oxidation products in the Health Professional Follow-up Study in controls (N = 460), 1993–1995. (DOCX 15 kb) [file 12894_2015_83_MOESM2_ESM.docx]

**Additional file 2: Table S2. Baseline characteristics according to tertiles of plasma fluorescent oxidation products in the Health Professional Follow-up Study in controls (N = 460), 1993-1995**

| **Variables** | **FlOP_360** | | | **FlOP_320** | | | **FlOP_400** | | |
| --- | --- | --- | --- | --- | --- | --- | --- | --- | --- |
| Tertile | 1 | 2 | 3 | 1 | 2 | 3 | 1 | 2 | 3 |
| Range (FI/ml) | < 184 | ≥ 184;  < 233 | ≥ 233 | < 356 | ≥ 356;  < 524 | ≥ 524 | < 49.1 | ≥ 49.1;  < 62.6 | ≥ 62.6 |
| N | 164 | 144 | 152 | 160 | 142 | 158 | 150 | 160 | 150 |
| Age (years) | **61.4** | **61.9** | **62.5** | **60.1** | **62.6** | **63.2** | 61.5 | 62.1 | 62.2 |
| Body mass index (kg/m^2^) | 25.8 | 26.1 | 25.9 | 25.5 | 26.0 | 26.3 | 25.6 | 26.0 | 26.1 |
| Alcohol intake (g/day)* | **2.1** | **8.8** | **9.6** | 2.6 | 9.8 | 8.6 | **2.1** | **6.6** | **10.3** |
| Physical activity (MET-hours/week)* | 28.8 | 37.0 | 29.3 | 28.9 | 37.5 | 28.4 | 28.3 | 36.8 | 27.7 |
| Caucasians (%) | **89** | **89** | **96** | 88 | 92 | 94 | 93 | 87 | 95 |
| Fasting status (≥ 8 hours; %) | **78.1** | **59.7** | **52.0** | **78.1** | **57.8** | **54.4** | **74.7** | **60.0** | **56.7** |
| History of BPH with surgery (%) | 2.4 | 4.2 | 3.3 | 1.9 | 2.8 | 5.1 | 2.7 | 3.8 | 3.3 |
| History of hypertension (%) | **25.6** | **25.7** | **35.5** | 22.5 | 33.8 | 31.0 | 23.3 | 33.8 | 29.3 |
| History of diabetes (%) | 3.7 | 2.8 | 4.6 | 1.9 | 4.2 | 5.1 | 2.0 | 3.8 | 5.3 |
| Current smokers (%) | **2.3** | **6.1** | **12.7** | 2.4 | 10.0 | 7.9 | **1.3** | **6.6** | **13.0** |
| Past smokers (%) | **42.2** | **52.3** | **64.2** | **43.2** | **56.1** | **59.2** | **45.0** | **48.9** | **64.4** |

Variables with normal distribution are shown in mean, unless otherwise specified. *Variables with skew distribution are shown in median. Abbreviations: FlOP = Fluorescent oxidation products, FI = Fluorescent intensity units, MET = Metabolic equivalent, BPH = Benign prostatic hyperplasia.

Bold-faced values indicate statistically significance at *P* < 0.05 across tertiles of FlOPs.
