# Supplementary material for: Association between plasma fluorescent oxidation products and erectile dysfunction: A prospective study
Source: BMC Urol. 2015 Aug 14;15:85. doi: 10.1186/s12894-015-0083-9 (PMC4536733; doi:10.1186/s12894-015-0083-9)
Supplement: Additional file 3: Table S3. — Baseline characteristics according to tertiles of plasma fluorescent oxidation products (FlOPs) (N = 1,000): cross-sectional analysis in the Health Professional Follow-up Study, 1993–1995. (DOCX 13 kb) [file 12894_2015_83_MOESM3_ESM.docx]

**Additional file 3: Table S3. Baseline characteristics according to tertiles of plasma fluorescent oxidation products (FlOPs) (N = 1,000): cross-sectional analysis in the Health Professional Follow-up Study, 1993-1995**

| **Variables** | **FlOP_360** | | | **FlOP_320** | | | **FlOP_400** | | |
| --- | --- | --- | --- | --- | --- | --- | --- | --- | --- |
| Tertile | 1 | 2 | 3 | 1 | 2 | 3 | 1 | 2 | 3 |
| Range (FI/ml) | < 184 | ≥ 184;  < 234 | ≥ 234 | < 356 | ≥ 356;  < 532 | ≥ 532 | < 49.2 | ≥ 49.2;  < 62.7 | ≥ 62.7 |
| N | 333 | 333 | 334 | 333 | 333 | 334 | 333 | 333 | 334 |
| Age (years) | **61.6** | **62.3** | **62.9** | **60.8** | **62.5** | **63.4** | **61.1** | **62.9** | **62.7** |
| Body mass index (kg/m^2^) | 25.8 | 25.9 | 25.7 | 25.5 | 25.9 | 26.0 | **25.4** | **25.9** | **26.0** |
| Alcohol intake (g/day)* | **3.04** | **7.80** | **10.16** | **3.47** | **9.04** | **8.80** | **2.78** | **7.70** | **10.48** |
| Physical activity (MET-hours/week)* | 27.4 | 31.0 | 29.1 | 27.9 | 33.9 | 25.4 | 27.4 | 30.2 | 27.0 |
| Caucasians (%) | 92 | 92 | 96 | 91 | 95 | 93 | 94 | 91 | 95 |
| Fasting status (≥ 8 hours; %) | **76.3** | **58.0** | **50.3** | **76.0** | **57.1** | **51.5** | **70.3** | **61.6** | **52.7** |
| History of BPH with surgery (%) | 2.70 | 3.60 | 3.29 | 2.70 | 3.30 | 3.59 | 3.00 | 3.90 | 2.69 |
| History of hypertension (%) | **23.1** | **22.5** | **33.5** | 19.5 | 30.6 | 29.0 | 21.6 | 31.2 | 26.4 |
| History of diabetes (%) | 3.60 | 3.30 | 3.29 | 3.00 | 3.30 | 3.89 | 3.00 | 3.90 | 3.29 |
| Current smokers (%) | **1.57** | **6.75** | **13.14** | 2.08 | 9.40 | 9.33 | **0.99** | **4.43** | **17.69** |
| Past smokers (%) | **39.2** | **50.0** | **59.8** | **39.4** | **55.2** | **54.4** | **35.8** | **50.0** | **63.6** |

Variables with normal distribution are shown in mean, unless otherwise specified. *Variables with skew distribution are shown in median. Abbreviations: FlOP = Fluorescent oxidation products, FI = Fluorescent intensity units, MET = Metabolic equivalent, BPH = Benign prostatic hyperplasia.

Bold-faced values indicate statistically significance at *P* < 0.05 across tertiles of FlOPs.
