# Supplementary material for: Association between plasma fluorescent oxidation products and erectile dysfunction: A prospective study
Source: BMC Urol. 2015 Aug 14;15:85. doi: 10.1186/s12894-015-0083-9 (PMC4536733; doi:10.1186/s12894-015-0083-9)
Supplement: Additional file 4: Table S4. — Association between plasma fluorescent oxidation products (FlOPs) and erectile dysfunction (N = 1,000): cross-sectional analysis in the Health Professional Follow-up Study, 1993–1995. (DOCX 15 kb) [file 12894_2015_83_MOESM4_ESM.docx]

**Additional file 4: Table S4. Association between plasma fluorescent oxidation products (FlOPs) and erectile dysfunction (N = 1,000): cross-sectional analysis in the Health Professional Follow-up Study, 1993-1995**

| **Tertile** | **1** | **2** | **3** | ***P* for trend** |
| --- | --- | --- | --- | --- |
| **Variables** | **FlOP_360** | | |  |
| Range (FI/ml) | < 184 | ≥ 184; < 234 | ≥ 234 | --- |
| N | 333 | 333 | 334 | --- |
| Median (FI/ml) | 161 | 207 | 282 | --- |
| Baseline erectile dysfunction (n, %) | 7 (2.1%) | 11 (3.3%) | 19 (5.7%) | --- |
| Age adjusted | 1 (ref) | 1.52 (0.57, 4.06) | **2.67 (1.09, 6.58)** | 0.03 |
| Multivariable adjusted* | 1 (ref) | 1.37 (0.49, 3.82) | **2.68 (1.01, 7.12)** | 0.03 |
|  | **FlOP_320** | | |  |
| Range (FI/ml) | < 356 | ≥ 356; < 532 | ≥ 532 | --- |
| N | 333 | 333 | 334 | --- |
| Median (FI/ml) | 304 | 414 | 1858 | --- |
| Baseline erectile dysfunction (n, %) | 8 (2.4%) | 10 (3.0%) | 19 (5.7%) | --- |
| Age adjusted | 1 (ref) | 1.03 (0.39, 2.70) | 1.88 (0.79, 4.45) | 0.08 |
| Multivariable adjusted* | 1 (ref) | 0.91 (0.33, 2.53) | 1.73 (0.68, 4.42) | 0.10 |
|  | **FlOP_400** | | |  |
| Range (FI/ml) | < 49.2 | ≥ 49.2; < 62.7 | ≥ 62.7 | --- |
| N | 333 | 333 | 334 | --- |
| Median (FI/ml) | 44.2 | 55.5 | 72.6 | --- |
| Baseline erectile dysfunction (n, %) | 6 (1.8%) | 16 (4.8%) | 15 (4.5%) | --- |
| Age adjusted | 1 (ref) | 2.03 (0.77, 5.39) | 2.13 (0.80, 5.65) | 0.23 |
| Multivariable adjusted* | 1 (ref) | 2.25 (0.80, 6.33) | 2.02 (0.70, 5.88) | 0.28 |

Values are odds ratio (95% confidence interval), unless otherwise specified. FI = Fluorescent intensity units.

*Risk factors include age (continuous), body mass index (continuous), alcohol intake (in quartiles: < 0.9, ≥ 0.9 and < 6.6, ≥ 6.6 and < 17.2, ≥ 17.2 g/day), physical activity (in quartiles: < 13.85, ≥ 13.85 and < 28.9, ≥ 28.9 and < 51.95, ≥ 51.95 MET-hours/week), Caucasian (yes/no), fasting hours (continuous), benign prostatic hyperplasia with surgery (yes/no), history of hypertension (yes/no), history of diabetes (yes/no), smoking status (current smokers, past smokers and non-smokers), month of blood draw (in seasons: Spring [March, April and May], Summer [June, July and August], Fall [September, October and November], Winter [December, January and February]) and year of blood draw (1993, 1994 and 1995).
